# Youth and professional perspectives of mental health resources across eight countries

**DOI:** 10.1016/j.childyouth.2022.106439

**Published:** 2022-05

**Authors:** Panos Vostanis, Florence Ruby, Jenna Jacob, Şeyda Eruyar, Elijah Mironga Getanda, Sadiyya Haffejee, Murali Krishna, Julian Edbrooke-Childs

**Affiliations:** a University of Leicester, School of Media, Communication and Sociology, University Road, Leicester, UK; b Anna Freud Centre, Child Outcomes Research Consortium, 4-8 Rodney Street, London N1 9JH, UK; c Necmettin Erbakan University, Department of Psychology, Köyceğiz 42140 Meram, Konya, Turkey; d Friendly Action Network Organization, Nacha Plazza, PO Box 13828-20100, Nakuru, Kenya; e University of Johannesburg, Auckland Park, Johannesburg 2092, South Africa; f FRAMe Mysore, New Kanthraj Urs Road, Kuvempunagar, Mysore 570023, India

**Keywords:** Youth, Global, Mental health, Support, Services, Stakeholders

## Abstract

**Introduction:**

Youth mental health support and services vary across sociocultural contexts. It is important to capture the perspectives of youth with lived experiences for planning needs-led interventions and services, especially in Global South Countries (GSC), with limited specialist resources and representative literature.

**Methods:**

The aim was to establish how youth with lived experiences of anxiety and depression viewed external support in different countries, and how these views were juxtaposed with those of professionals. We involved 121 youth aged 14–24 years and 62 professionals from different disciplines in eight countries, predominantly from the Global South. Two youth and one professional focus group was facilitated in each country. The data were analysed through a codebook thematic approach.

**Results:**

Youth across all countries largely valued informal support from family, peers and community, whilst those from GSC had limited access to structural support. They related lived experiences to therapeutic engagement and processes, in contrast with professionals who focused on outcomes and service delivery. Mental health awareness and integration of interventions with social support were considered essential by both youth and professionals, especially in disadvantaged communities.

**Conclusion:**

The mental health needs of youth in disadvantaged GSC communities can be best met through multi-modal interventions addressing these needs across their socioecology and positioned within a stepped care model. Youth with lived experiences should be involved in service planning, implementation and monitoring.

## Highlights

• Youth mental health support in Global South Countries should be concurrently addressed at the inter-linked relational, systems and structural levels.
• Forming meaningful connections are central to accessing such support.
• Youth with mental health lived experience should be incorporated in the planning, implementation and evaluation of services.

## 1 Introduction

Child and youth mental health is a priority in international and local policy (Patel et al., 2018, United Nations, 2014). There is both recognition and emerging evidence of the impact of enduring mental health problems on several domains of life quality and future outcomes (Erskine et al., 2015). Mental health difficulties account for 16% of the global burden of disease and injury in youth 10–19 years; whilst half of all mental health conditions start by 14 years of age (World Health Organization, 2020). Depression is a prominent and increasing contributor to global health burden, and has adverse impact on individual, familial, community, economic and health outcomes (Kutcher et al., 2019). Providing adequate mental health interventions is thus now a priority in terms of Sustainable Development Goals (SDGs - United Nations, 2015).

Globally, there are broad similarities in underpinning risk and protective mechanisms, although these are often influenced by cultural and other contextual factors (Srinath et al., 2010, Theron and Liebenberg, 2015). Major differences lie in service provision, with substantive inequalities between Global North Countries (GNC) and Global South Countries (GSC) in terms of infrastructure, specialist resources and skilled staff (World Health Organization, 2018). This gap is accentuated by socioeconomic disadvantage, stigma of mental illness, and lack of culturally appropriate interventions (Cullins and Mian, 2015, Getanda et al., 2017). Mental health support, however, can be extended beyond specialist services to family and community networks, schools, religious groups and social activities, which are often more readily available in GSC. These resources have been found to provide greater accessibility and acceptability, trust, and family engagement (Kohrt et al., 2018).

As youth with mental health difficulties are increasingly at the centre of service design and delivery, it is important to capture their lived experiences of support across different sociocultural contexts and systems. Most evidence is based on GNC studies on help-seeking. Youth have been shown to value ease of access, age-friendly approaches and environments, flexibility, building a relationship with professionals, being listened to, receiving individualized care plans, and being empowered as active agents in decisions about their care (Frauenholtz and Menderhall, 2020, Loughhead et al., 2018). Youth usually feel more comfortable in first seeking informal support such as talking to family, friends and teachers, and/or looking for information by themselves (Heerde & Hemphill, 2018). These preferences centre on their understanding of mental health, fears of being judged, need for confidentiality, and a wish to be self-reliant (Del Mauro & Jackson-Williams, 2013).

In contrast, attributing mental health difficulties to personal causes, structural barriers and negative beliefs of professional sources can act as deterrents to help-seeking (Velasco et al., 2020). Other negative mental health experiences include lack of information on how to access services, being compelled to attend therapy by parents and carers or teachers, lengthy waiting times, poor communication, and staff changes (Persson et al., 2017).

There is less evidence on youth perspectives of mental health support systems in GSC. Available evidence suggests that conceptualization of mental health or illness (Tamburrino et al., 2020) and fears of negative societal attitudes (Khalil et al., 2020) are prominent barriers to help-seeking. This evidence also shows that available informal and formal support usually consist of extended family, community forums and networks, schools, primary health clinics, social and sports activities (Clark et al., 2018, Panigrani et al., 2018). Indeed, youth in four GSC were found to utilize internal and informal rather than formal and structural resources when faced with trauma-inducing scenarios (Vostanis et al., 2020). If services are to engage and actively involve youth, their perspectives need to be understood in conjunction with those of professionals and other providers across a range of cultures and systems.

### 1.1 Study orientation

In GSC, where specialist resources are sparse, a dynamic interrelatedness among personal and environmental factors, including the family, school, community and mental health agencies is essential in addressing youth mental health difficulties (Reupert, 2017, Vostanis, 2017). For this reason, the study was conceptually informed by the socioecological paradigm, which places youth mental health service needs along several dynamically connected domains (Bronfenbrenner, 1979).

At a service level, this research was also informed by the stepped care model of care provision (World Health Organization, 2014). This is endorsed by public health and welfare policy across systems, and for a range of mental health difficulties. It is particularly pertinent for youth mental health care, because of the importance of involving caregivers, schools and other frontline agencies at various stages of help-seeking, for example, for common mental health difficulties like anxiety (Ollendick et al., 2018). In GSC, first-level response through school and community resources has been shown to be even more important than GNC in terms of engagement, participation and costs such as in post-disaster (McDermott & Cobham, 2014) or refugee contexts (UNHCR, 2013).

Policy and research gaps in understanding youth experiences of mental health support across sociocultural contexts and public healthcare systems informed the rationale for this study. The broad aim was thus to establish youth and professionals’ perspectives and experiences of external informal and formal support for common youth mental health difficulties (depression and anxiety) across different cultural contexts and service systems. In particular, the study addressed the following research questions:

a. How do youth with lived experiences of depression and anxiety perceive external mental health support (current and recommended) across eight countries?

b. How are these youth perceptions juxtaposed with those of professionals from different disciplines operating in the respective areas?

The research questions were explored through a qualitative design involving youth and professionals in focus groups discussions in eight countries, as described below.

## 2 Materials and methods

### 2.1 Context and participants

Participatory Action Research (PAR; Baum et al., 2006) emphasizes the collaborative participation of researchers and the community with lived experience (Higginbottom and Liamputtong, 2017, Kindon et al., 2007). Participants work collaboratively alongside researchers to develop actions and knowledge collectively. PAR is increasingly used to cooperatively address problems affecting individuals who are marginalized or excluded from service planning and implementation such as youth. (Rhodes et al., 2012). This approach allows for active involvement of youth throughout the research process, reducing power imbalances and enabling spaces to be heard (Flewitt et al., 2018, Liebenberg et al., 2020). PAR was considered the appropriate approach to the present research, ensuring that young people were centrally involved in the design and delivery of this project, giving prominence to their voices, which is fundamental to the exploration of the research questions. A qualitative research design was appropriate to investigate the main aim of the research: to establish how youth with lived experiences of anxiety and depression viewed external support in different countries, and how these views were juxtaposed with those of professionals. This design is an appropriate fit to give depth and context to the exploration of the views of youth and professionals, which might otherwise not be possible.

We selected countries that were broadly representative of the socioeconomic spectrum across the Global South and North (OECD, 2016). These consisted of India, Pakistan, Turkey, Kenya, South Africa, Portugal, United Kingdom and Brazil. Although we could have confined the sample to GSC, because of the research gap and population needs, we opted to include youth experiences and perspectives from two GNCs too, in order to explore both commonalities and context-specific issues across different systems. The sampling procedure reflected such system-related differences on the ground. Within each country, a non-governmental organization (NGO – six countries: Brazil, Kenya, South Africa, Turkey, India and Pakistan) or academic institution (Portugal) or peer-led lived experience charity (UK) acted as local project lead. These lead agencies were identified through existing global youth mental health networks by the central research team (Vostanis, 2019). At the next stage, we adopted a purposive sampling strategy.

Each host agency, through their local networks (face-to-face and via email), invited young people aged 14–24 years who had experienced depression or anxiety, defined as a combination of symptoms (such as pervasive low mood, irritability, impaired sleep or appetite, or physiological presentations of anxiety) that had an impact on their everyday life functioning. Depression and anxiety were selected as common mental health difficulties, especially during adolescence and young adulthood.

The host agency subsequently invited professionals operating with this age group in their area. As already evidenced by the literature (e.g., Patel et al., 2018), we anticipated limited availability of specialist mental health services in GSC. For this reason, professionals were selected in relation to a broader definition of youth psychosocial needs, i.e., from welfare, education, health and community or religious-based agencies. Practitioners employed in a generic role had a professional qualification in education, social work or psychology. Hence, they are referred to as NGO or Youth Workers in the results section. Researchers and policy makers were also invited, if working in the same field. The target was to involve on average 6–8 participants per focus group. Two youth and one professional focus group were facilitated in each country. In total, 121 youth and 62 professionals took part in the study. This size was considered as likely to achieve thematic saturation across the youth and professionals samples, although not necessarily within each site (Hancock et al., 2016), and to comply with study logistics and resources. The participants’ profile is presented in Table 1. Overall, there was a higher ratio of female youth and professionals involved. Also, there was a degree of self-selection in high proportion of youth being college or higher education students.

**Table 1:** Participants’ profile (youth n = 121, professionals n = 62).

Country / Site	Youth Focus Group 1	Youth Focus Group 2	Professionals Focus Group - Role
Brazil	n = 6, 14–17 years (mean 15)	n = 5, 22–23 years (mean 23)	n = 6 Psychologist (2) Social articulator Social assistance co-ordinator Shelter co-ordinator Education co-ordinator
India	n = 6, 18–24 years	n = 7, 18–24 years	n = 9 Speech therapist Occupational therapist Social worker Psychologist Psychiatrist Physician Clinical researcher (2) Academic
Kenya	n = 8, 14–18 years (mean 16)	n = 8, 20–24 years (mean 21)	n = 6 Religious leader NGO worker School principal Educational counsellor Social worker Psychologist
Pakistan	n = 11, 18–24 years	n = 12, 18–24 years	n = 9 Educational counsellor (3) Teacher Psychologist (4) Academic
Portugal	n = 6, 19–24 years (mean 21)	n = 8, 17–24 years (mean 20)	n = 10 Psychologist (6) Psychotherapist (3) Researcher (1)
South Africa	n = 7, 17–22 years (mean 20)	n = 7, 18–23 years (mean 20)	n = 7 Counsellor Psychotherapist Psychologist Youth worker (sports programme) Youth skills facilitator NGO manager (2)
Turkey	n = 8, 18–24 years	n = 8, 16–24 years	n = 8 Psychologist (5) Social worker (2) Researcher (1)
United Kingdom	n = 7 (18–24 years)	n = 7 (18–24 years)	n = 7 Psychologist (2) Academic/Policy (4) Other

## 3 Research procedure

Research ethics approval was obtained by the University of Leicester Psychology Research Ethics Committee in the UK. Youth aged 16–24 years and professionals provided written informed consent. Parents or carers of younger participants aged 14–16 years gave written informed consent, following which youth provided verbal assent.

Focus groups were considered the most appropriate approach of addressing the research questions by engaging different stakeholder groups in ‘collective conversations’ (Liamputtong, 2011) in relation to their experiences, insights and perspectives (Onwuegbuzie et al., 2009). The focus group topic guide was informed by the literature on factors involved in the development of depression and anxiety, range of interventions, as well as systems across a young person’s socioecology. In particular, youth were asked about their lived experiences, support they had found helpful (and reasons for it), barriers and challenges, as well as recommendations on how support could be improved in the future. Participants were asked to provide examples to support their statements and views, where appropriate. Professionals were asked to explore the same issues from their perspective and within their agency role (for example, school, NGO or mental health setting). Each youth focus group was moderated by a senior member of the host organization, with co-moderation by a peer researcher. Although all moderators had previous experience of facilitating focus groups, additional training was provided by the research team in relation to the aims of this study. This training, in conjunction with the involvement of peer researchers, particularly aimed to balance and safeguard the researcher-youth participant relationship. A member of the central research team observed remotely the focus group discussions.

As the study was conducted during the COVID-19 pandemic, facilitation was influenced by national and local safety guidelines. The majority of focus groups were conducted face-to-face, with a small number being conducted remotely on the Zoom digital platform. The duration of each focus group was approximately 90 min. Focus groups were facilitated in the local language and were audio-recorded. These were transcribed and translated into English, following which the overall dataset was coded.

This research design involved different levels of ‘insider-outsider’ challenges (Kanuha, 2000), in contrast with studies conducted in a single site and/or with a relatively homogenous group such youth or parents only. The central research team could thus be viewed as external by different participating countries; whilst certain partner countries (e.g., from Global North) could be viewed as external by Global South participants. Local senior researchers could be viewed as insiders in terms of context and cultural knowledge, whilst being outsiders in relation to experiences of being young and living in disadvantage. In contrast, peer researchers could be viewed as insiders by youth but as inexperienced outsiders by professionals. In order to address these methodological issues through the research (planning, data collection, analysis and interpretation), we adopted Dwyer and Buckle’s (2009) positionality of researchers at all levels ‘creating space’ to function as ‘both’ outsiders and insiders, instead of a dichotomous ‘either or’ approach. This integration was ensured by aligning to qualitative methods criteria, especially sincerity (self-reflexivity and transparency) and credibility (such as member reflections and multivocality) (Tracy, 2010) during the regular communication forums between central research team, local senior and peer researchers.

### 3.1 Data analysis

Thematic analysis was the adopted framework in identifying, analysing and reporting patterns (themes) within the data (Braun & Clarke, 2006). This was congruent with the open nature of the research questions, and the goal of identifying common issues at stake across countries and perspectives. Both inductive and deductive methods were utilized, as the researchers had not adopted a pre-existing coding frame while searching for new concepts in the dataset, yet identified the themes related to the research questions and the wider literature in conjunction with the data. This combination of inductive and deductive, structured and unstructured approach to conceptualization and identification of categories within the data, as underpinned by the broader literature and goals of the research project, is congruent with a ‘codebook’ approach to thematic analysis (Braun and Clarke, 2019, Clarke and Braun, 2018).

The codebook approach was thus the method of choice, as it uses a combination of inductive and deductive analysis, whereby the coding is participant-driven, but utilizes the structure of the interview guide and wider evidence, where relevant, to facilitate the creation and organization of the codes. This deductive aspect can also help with the labelling of certain codes, where there is uncertainty. The data was initially coded by one researcher as part of a larger project, and it was revisited by a second coder in relation to the research questions of this study. Two further independent research team members helped resolve any discrepancies. This process thus assured cohesion across coding and results in coder agreement. A youth advisory group, consisting of 1–2 representatives from each country, met on three occasions throughout the project to advise on the design, recruitment, contextual representation of participants’ contributions, and interpretation of the data, thus provide youth-centric supplemental checks to the analysis. The research team held regular internal meetings and meetings with partners from participating countries to ensure consistency and integrity in data collection and analysis. All co-authors finally checked that the interpretation of the data in the codebook was consistent within their cultural context.

## 4 Results

The analysis led to three superordinate themes and 14 subthemes. These are

summarized in Table 2 and described in detail below. Supporting excerpts are provided. Where statements on a finding relate to several excerpts that could not be included in the manuscript, this is explicitly stated. Some statements are based on several excerpts (for example, on the use of different therapeutic modalities), for which reason selected quotes are included below. The juxtaposition between youth and professionals is highlighted when there are discrepancies in their accounts, or when a subtheme is raised by only one stakeholder group.

**Table 2:** Themes and subthemes on youth mental supports in eight countries (youth n = 121 and professionals n = 62).

Themes	Subthemes
Relational	Family relationships Social connections Community engagement Creative expression Religious and faith-based practice
Informal systems	Connection with environment and nature Promotion of awareness Interconnecting systems Role of social media
Formal structural	Addressing disadvantage Therapeutic modalities Pharmacological interventions Joined-up approach Preventive strategies

### 4.1 Theme 1: Relational supports

The family was variably viewed as a source of nurturing, trust, understanding and good communication by youth in GSC, although rarely mentioned by professionals. Youth in GSC referred to extended family relationships such as with grandparents and cousins and related to broader social connections. Youth acknowledged that the family can also put pressure on them through expectations, incompatible beliefs, lack of availability, or conflict; for example, through shouting, all of which were described as potentially leading to or exacerbating mental health difficulties. Parents’ and carers’ perspectives on mental health difficulties such as depression often shaped their responses and were hence inked to the later subtheme of raising awareness.

*“So, when this…when this…maybe this brother…this small brother will tend to uplift the older brother to show him that you actually matter in the things of this family. The fact that there is increased sense of mattering will reduce depression for this one.”*

Focus group 1, Youth 4, Kenya

*“The parents see the child inadequate and say he should be more perfect. So, the child also does not accept himself in this way, he sees himself inadequate. He tries hard to achieve things that cannot be done, or the family expect impossible things.”*

Focus group 1, Youth 2, Turkey

Social connections, especially with peers but also neighbours, were highlighted by all groups. Youth mentioned several mechanisms such as sense of belonging, offloading, listening, sharing, problem-solving, and caring for others; most of which are non-specific therapeutic factors in different modalities. Some youth participants made a distinction between being able to connect (or re-connect) rather than merely interacting or mixing socially. Professionals focused more on outcomes rather than ‘ingredients’ of social connections such as reducing isolation, learning how to build relationships and how to manage their ‘social capital’. Potential counter-productive effects were raised such as being exposed to ‘negativity’, feeling intimidated or hurt, and drawing comparisons with peers.

*“I end up absorbing more stories and experiences that generate connections in my head and help me solve problems. I like to listen to and to share ideas while socializing, but then I prefer to deal with my problems on my own.”*

Focus group 1, Youth 2, Brazil

*“But then, I see a difference between connection and relationships. Like you can have a lot of social connections without them being necessarily good, solid relationships.”*

Focus group 1, Youth 4, UK

Similar benefits were generated for the next outer level of engaging with the wider community, i.e., relational aspects rather than activities per se. Neighbourhood cohesion and closeness were reported by GSC participants, although these interestingly also increased in GNC through the COVID-19 pandemic, including intergenerational contact. Living in rural or city areas often determined the extent of neighbourhood cohesion, especially among disadvantaged communities. Feeling safe, accepted and belonging were reported by youth. Some youth participants from Turkey, India and Pakistan cautioned that supportive community structures can also reinforce stereotypes and increase pressures, mainly on young women. Youth and professionals made recommendations on initiating community engagement through volunteering and life skills projects.

***“****…the rural people, they already have communication with the neighbours, and they enjoy their neighbourhood well. When it comes to urban places, it’s really not usual things…like in the urban, people are like, they mind their own business, they don’t mingle at all…and when they meet, they don’t talk openly about anything. So, in urban places, if people improve their neighbourhood cohesion, it is definitely helpful for them.”*

Focus group 2, Youth 2, India

“… it seems to me that, in some respects, this is far from some Brazilian realities. It would be perfect if it happened, but I think it is important to tell teenagers and young people that, even in the absence of a cohesive neighbourhood, all these other ingredients are available.”

NGO worker, Brazil

Creative expression in a social context was viewed as particularly helpful to youth struggling at school, for example through drama, music, theatre and sports (with several excerpts relating to respective modalities). Both youth and professionals valued creative expression, whilst professionals related creative modalities to interventions and service provision. Schools were viewed as central in creating opportunities to support all youth and to intervene for those with mental health difficulties by making resources available. Described processes provided an escape from adversities, and assisted in stress reduction, gaining confidence, stimulation of social life, and resulting social benefits. When not carefully planned or matched to youth needs, however, involvement as an actor or spectator could compound anxiety.

“When we use music training or any tapping technique, it is not exactly music training. Even the simple tapping technique, will reduce your stress.”

Speech and language therapist, India

As in previous research, individual resources of religious coping and religiosity or spirituality were often intertwined with religious-based practice shared with others. In this relational context, the role of religious affiliations was highlighted in creating a sense of belonging. This was particularly reported by professionals rather than youth, and from Islamic Asia countries (Turkey and Pakistan), with some excerpts originating from Christian Africa countries (South Africa and Kenya). Religion- or faith-based resources were extended to acquiring spirituality, humanity, values or a sense of responsibility. Some connections were also made with therapeutic processes of instilling hope and problem-solving.

“*Religion is hope for a lot of these kids. They take it as, you know, we have the hope and these prayers they are like, you know, it’s something we can hold it onto, so probably that is it’s about faith. It’s about the mindset…and they really believe if they're going to do this, is going to solve their problems. So, what thinking and thinking that is how prayer and all of these things were taken, as you know, if you're going to do with full belief is going to help us. So, positive psychology.”*

Psychologist, Pakistan

### 4.2 Theme 2: Informal systems support

Participants across countries and stakeholder groups appreciated connectivity to nature and the environment, and related it to forming social connections (e.g., through activities) and community engagement. Nature was thus seen as inter-linked with other relational support. It was also linked to individual coping strategies (which are not the focus of this paper) such as attributing meaning, relaxation, and imagery (*“placing oneself somewhere else”*). Opportunities to access green spaces were, however, compromised in city areas, especially for disadvantaged communities.

“Whenever I experience something bad in my life, I immediately buy a flower, a soil, a pot and take care of it, seeing it grow seems like a reason to live.”

Focus group 1, Youth 2, Turkey

Youth participants acknowledged that environmental values were not widely appreciated across society. Limited awareness was described as a challenge in related topics such as vulnerability, emotional literacy and, crucially, mental health. Stigma of mental illness across family, community and societal systems was viewed as still largely prevalent, related to fears and myths, and acting as barrier to help-seeking.

“*I think that we have to demystify these situations and how society thinks… because, sometimes there are some clues, but people are afraid of talking about their problems due to their own preconceptions or the lack of education about mental health. Even parents who do not know how to deal with children with anxiety or depression problems, and do not know if they should take them to a consultation, should be more educated about mental health problems.”*

Focus group 2, Youth 3, Portugal

Suggestions among youth and professionals across countries started with challenging and changing attitudes within families, communities and wider society, by normalizing mental illness and being non-judgemental. Stakeholders suggested that promotion programmes should target everyone, including youth, adults, parents/carers and professionals such as teachers. Youth with lived experiences conveyed that they should be empowered to challenge negative attitudes and promote awareness. Some professionals extended the adverse impact of stigma to marginalized groups through labelling and misinterpreting emotional needs for externalizing behaviours.

*“I think in our society, people suffering from depression get to hear a lot from the outer world. Like, you’re being ungrateful, or you should be happy with what you have. I think people suffering from depression should learn to say ‘no’ or should learn to tell them to stop what they are saying.”*

Focus group 2, Youth 3, Pakistan

*“…those children did not arrive in a vacuum, and that protest is so loud, we just don’t hear it. Because the behavior is such a protest that we send them to court and to all kinds of interesting things, except to listening to them and saying to them ‘what world did we hand you that you have to protest so hard against’?”*

Youth worker, South Africa

Youth participants valued building relationships with professionals in different contexts. Youth raised a range of processes of non-specified professional contact such as talking, sharing emotions, receiving tools to cope better, having space, and re-gaining control in decisions about their life.

*“Therapists should also understand and legitimize our feelings and be able to show us that we have the power to face these kind of situations and turn things around. Young people with difficulties should also make an effort to turn things around and start feeling better.”*

Focus group 1, Youth 1, Portugal

Social and conventional media were considered as powerful tools that are routinely used by youth. Potential was raised for awareness, psychoeducation, building coping strategies (such as humor), and following role models, especially those who shared similar experiences. Risks were also acknowledged in role models setting stereotypes of fictional and superficial expectations of happiness and healthy lifestyles to youth. Youth participants mentioned both being able to form social connections and feeling lonely through social media platforms.

*“Nowadays, everyone is addicted to mobiles, and there are some apps like Facebook, Instagram, in that social platforms many people show that, they are perfect, they have perfect family, car…like they have a beautiful, perfect life. In that not everyone is perfect, so, being frustrated that I am not perfect is not so cool. So, reducing perfectionism is important in preventing depression and anxiety.”*

Focus group 1, Youth 2, India

### 4.3 Theme 3: Formal structural support

Particular attention was given by all focus groups to youth living in disadvantage, and how their multiple needs, which are often compounded by poverty and inequalities, can be addressed. Even in GNC, increasing numbers of youth were faced with adversity and unemployment in the aftermath of the COVID-19 pandemic. Although there was consensus on the importance of targeting resources in conjunction with psychosocial interventions, there were mixed views, across youth and professionals, on what these should entail. Addressing basic needs such as nutrition, housing and school uniforms were believed to be clearly paramount.

It was acknowledged that financial incentives could help access psychosocial resources such as social activities and better quality of care, as secondary education and healthcare are not free in many GSC. These could also help if depression was a direct consequence of financial issues such as losing one’s job. However, even in these circumstances, participants suggested that financial support should be short-term, and linked to long-term stability and safety, by investing in education and life skills. Otherwise, there was a risk of fostering dependence and wasting resources in material goods, thus diminishing self-esteem further. Youth participation in making complex choices was viewed as important in their future ownership and engagement.

*“…to give them food…and give them a home stay, where they could have the space to play that they are not going to be kicked out after two months. So, that would be for me the first one…and give them a uniform, so that they don’t feel out, you know, give them the schoolbook and…uhm…you know, for me a lot of it in the environment is not so much in the child, it’s in the environment.”*

Psychologist, South Africa

*“…increased opportunities for involving young people in that change making process.”*

Focus group 1, Youth 3, UK

As anticipated from the literature, variation in perspectives between GSC and GNC participants largely emerged in experiences of services. Youth mental health services were mentioned by youth from Portugal and the UK, and by mental health professionals (psychiatrists or psychologists), e.g., from India and Turkey. Overall, youth mental health provision was either absent or not easily available.

Nevertheless, participants widely referred to different types of therapeutic modalities or their applications in schools and community settings. The term ‘counselling’ was used loosely by stakeholders. This was positively perceived, even in contexts with limited access to talking therapies. Youth who had received professional support felt they had benefited from acknowledging the problem, challenging misconceptions on mental health, shifting negative mood, gaining confidence and strength, and understanding oneself.

*“…for example, I had been to counselling, I talked about sadness and happiness…it was one-hour counselling…. when I came outside, within five minutes, I was happy a lot, lot of happiness was there…they will do something, mind will be relaxed…I got courage.”*

Focus group 1, Youth 3, India

Several types of interventions were listed, especially by professionals, across all countries. These included mindfulness, relaxation and yoga (predominantly by professionals and youth in India and Pakistan); creative therapies (art, music, psychodrama); cognitive-behavioural therapy (CBT); alternative therapies (interestingly, mainly sought for in urban centres); and group work – these were supported by several excerpts not included below. Youth tended to describe the processes through which they were helped, and which were similar to the non-specific elements described under the previous subtheme, largely in terms of dealing with stressors; whilst professionals related to their delivery.

*“…these relaxation techniques for, e.g., deep breathing, helps, which focuses on body and brings us to the present. I personally tried deep breathing exercise to focus on my present, and sometimes it takes you out from worries.”*

Focus group 1, Youth 10, Pakistan

*“When I keep on handling learning disabled kids, they usually experience more of stress in this scenario… relaxation, mindfulness, and also proper counselling matters here.”*

Speech and language therapist, India

A UK youth participant cautioned against rigid implementation, for example of family therapy, without prior engagement.

…where you have to actively change, not just the way you see things, but also how you think about things, and adopting new patterns or new methods.”

Focus group 2, Youth 3, UK

Views on the indications for pharmacological treatment provided by different health services, mainly through antidepressants, were mixed across and within stakeholder groups and countries. Many youth participants appeared influenced by their own experiences, as well as by family and friends. Concerns included dependence, not addressing underlying problems and side-effects. Stigma was more prominent in GSC. Indications for the use of antidepressants included symptom severity, crisis management and monitoring, and combination with psychological approaches, preferably as the last resort.

*“There are people who can’t survive without these (antidepressants) because they…like…over relied on it. So, when they get in…before they get into depression, they take the antidepressants to help their bodies cool down…so, on scenarios where you get people using antidepressants, so as to prevent depression, there is a challenge of overreliance and overdependence. And also, the issue of addiction comes in. So, when we really encourage the youth bracket, maybe to use antidepressants as a solution, that won’t be the case. Let it be the last solution.”*

Focus group 1, Youth 2, Kenya

*“I think the use of antidepressants is somewhat effective. There are people who find it effective and others who don’t.”*

Focus group 2, Youth 5, Brazil

Interestingly, several participants, across stakeholder groups and countries, valued the adoption of a holistic approach in mental health care (mainly supported by youth participants), and in joint working between agencies on the ground (mainly supported by professionals). This approach should reflect the multiple factors involved in the development of mental health difficulties, hence the complexity of youth mental health needs.

“*First of all, talk to your family, because they are the one who can bring you out from depression, they know you well. Friends might not know you well. Or you can talk to someone to whom you trust that, yes, she will help if you are not telling the family. Or you talk to psychologist” (Youth 4) … “I think all of them can be under one name that is communication” (Youth 7).*

Focus group 2, Pakistan

Finally, prevention was intermittently brought up throughout group discussions, albeit without consensus on its definition, or positioning within individual care or systems. Youth positioned prevention within previously considered support outside structural services, notably social connections. Several professionals proposed a continuum from prevention to treatment. Social and emotional literacy (SEL), psychoeducation, advocacy and peer support were specific preventive strategies mentioned by professionals, with schools being best placed for their implementation.

“Also, teaching the coping mechanisms as well, and how to understand and better help peers. So, basically, learn peer support in schools, so that we can all help each other through difficult times.”

Focus group 1, Youth 4, UK

*“And the preventive part that you asked, especially people who are into yoga, relaxation, meditation, and undergone this kind of training as a regular curriculum, or their daily routine lifestyle, they tend to function better and also handle stress better in their careers.”*

Psychiatrist, India

## 5 Discussion

The aim of this study was to capture the perspectives of youth with lived mental health experiences and those of professionals, on external support systems across different sociocultural contexts. Understanding these perspectives is important for tapping into existing resources, designing services and, notably, linking informal and formal supports in a seamless model that is also tailored to local and individual needs. Although this was an exploratory rather than comparative design, some findings could be attributed to youth rather than professionals, apply across the sample, vary between GNC and GSC, or be more relevant to certain societies.

All identified support was considered relevant to participants, albeit with variable weight and positioning within their systems. A key finding was the inter-dependence between different levels, which is consistent with the socioecological framework and the importance of multi-modal interventions (Vostanis, 2017), especially in communities of deprivation, where youth are more likely to present with multiple needs (Bornheimer et al., 2018). Context and cultural relativity are important, as what may be seen as not coping or 'dysfunctional' in one context may indicate resilience in another. For example, the use of medication may be seen as appropriate and necessary in one context, but in another it may be viewed negatively and discouraged. All these complex connotations are important in engaging youth and families to interventions and services that are both culturally and developmentally sensitive and engaging.

Recurrent references to various ‘connections’ (mesosystems) was an important finding. These were linked to individual resources (not addressed in this paper), micro (family, school and friends), exo (indirect environment, neighbours and media), macro (social and cultural values), and chrono systems (changes over time). What appeared to matter to youth participants was the meaning, quality and emotional impact of various supports, rather than merely having access to them. The sense of being able to form connections came across in positive experiences with professionals.

Overall, the findings are consistent with previous child and youth mental health literature in GSC, which largely originates from adult reports in different sociocultural contexts. As previously established, youth with mental health needs and living in GSC communities predominantly rely on informal than external structural support (Getanda et al., 2017, Patel et al., 2018). Access to limited resources is hindered by stigma among families and communities (Mackenzie et al., 2019), and the concentration of resources in urban centres for those youth with severe and entrenched disorders (Kamau et al., 2017). Engagement is further compounded by mistrust, and youth not being listened to, or sharing decisions about their care (Frauenholtz & Mendelhall, 2020); these factors are well established globally by help-seeking research. Connections and functionality were highlighted in providing material assistance that should hierarchically meet basic needs, whilst equipping youth with adaptive life skills in a socially inclusive manner (Kempe, 2012).

It is interesting to note subthemes predominantly mentioned by youth and professionals respectively, and how their often-complementary expertise can be utilized in service provision. Youth focused more on the role of the family, social media and therapeutic elements of contacts with professionals. Professionals predominantly highlighted the role of religious support, incorporation of interventions in service delivery, and outcomes. Some subthemes were raised by both groups but were approached from different perspectives. In particular, youth valued components of the therapeutic process (such as creative expression), whilst professionals focused on their incorporation in programmes (such as creative activities). Both groups highlighted the importance of meeting basic needs, incorporating practical and therapeutic support, improving environmental quality, and enhancing life skills through community projects in disadvantaged areas. Although such perspectives can be incorporated in service provision, they also indicate that professionals may not be aware of certain youth preferences and priorities. This discrepancy can be addressed by involving youth with lived experiences in staff training and youth-centric service planning.

### 5.1 Implications for policy and service provision

The inter-connectedness between vulnerabilities and mental health needs along the youth socioecology highlights the importance of integrating policy, service delivery and practice. Youth mental health policy should be linked to other youth-related sectors such as safeguarding, welfare, physical health, education, leisure, environment, and community regeneration. Policy should be reflected on the ground through interdisciplinary networks, joint working and training that maximize resources, in particular in GSC and in the absence of extensive specialist services. When provided with an interdisciplinary training framework, GSC stakeholders valued its principles and benefits (Vostanis et al., 2018). Capacity-building should be extended to community workers, religious scholars and volunteers (paraprofessionals), who have an important role in GSC in engaging youth and families, and delivering interventions (van Ginneken et al., 2013). Youth themselves have been shown to actively contribute to mental health care as peer educators or mentors (Cobbett et al., 2013). All these stakeholder groups could be incorporated in a stepped care model (World Health Organization, 2014).

Community awareness is essential in challenging and reframing personal, societal, organizational and professional stigma of mental health. This was acknowledged by both youth and professionals. Awareness campaigns and programmes can particularly benefit from the active involvement of youth with lived experiences, with buy-in from the community and religious leaders. Parental knowledge and beliefs are often compounded by economic hardship and service barriers such as transport in hindering help-seeking (Evans, 2010). Additional strategies thus need to be put in place to involve parents and make psychosocial support more accessible, for example, through community and school-based interventions.

As ‘counselling’ was variably and loosely referred to by participants, this therapeutic concept needs specification for different service levels and contexts, for example by differentiating between pastoral care, school counselling and trauma-informed approaches. The origins of certain psychological modalities like mindfulness and yoga in Eastern GSC offer opportunities for cross-cultural fertilization and application for both preventive and responsive purposes. Youth participants favoured the use of digital technology, which has been shown to enhance knowledge, engagement and intervention uptake (Fairburn & Patel, 2018).

### 5.2 Limitation

The findings should be interpreted in the context of certain methodological limitations. The sample of youth and professional participants was not necessarily representative of other countries, indeed of different socioeconomic, cultural or ethnic groups within the participating countries. Although youth participants shared unique living experiences, there was possibly a self-selection of more motivated and articulate students. It is well established, for example, that disadvantaged youth groups often have lower access to available sources of help, and correspondingly have more negative and stigmatizing experiences (Bringewatt & Gershoff, 2010). We did not collect additional sociodemographic characteristics to enable further analysis. Sampling adequacy was assured *across* but not necessarily *within* participating sites. We did not include parents or carers in the sample. As our youth participants’ age crossed from adolescence to young adulthood, more developmentally focused studies, including those with children, would provide evidence of how accessing support may vary at different life stages. Finally, further emphasis should be placed on understanding the personalization of available support, i.e., *what* does and does not work for *whom*, and under *what* circumstances.

It would be interesting for future research to map services within each country and to contrast youth and professionals’ perceptions against service activity data. Nevertheless, this study provides a unique cross-cultural reflection of how youth with experiences of depression and anxiety view support systems, interventions and services within and across countries; as well as how these views are juxtaposed with those of interprofessional groups operating in the same areas.

## 6 Conclusion

In the light of these findings, the contexts and systems within the young person’s own family, community, society and culture need to be considered when seeking the best ways to support them. This is demonstrated at several levels from our analysis: micro or relational systems (family, school and friends); mesosystems of connections; exosystems of indirect environment, neighbours, and media; macrosystems of social and cultural values; and chronosystems of changes in concepts, attitudes and services over time. Strategies should dynamically address and incorporate these systems. Multimodal interventions can integrate available approaches and staff competencies in GSC to meet complex youth mental health needs, especially in disadvantaged communities. Positioning such interventions within a stepped care model can hierarchically meet needs and maximize resources on the ground. Service provision should be supported by policy, interdisciplinary networks and capacity-building. Youth with lived experiences can contribute their unique knowledge to promote awareness, help-seeking and engagement through active involvement in service planning.

### CRediT authorship contribution statement

**Panos Vostanis:** Conceptualization, Formal analysis. **Florence Ruby:** Data curation, Methodology, Formal analysis, Investigation, Project administration. **Jenna Jacob:** Conceptualization, Funding acquisition, Project administration. **Şeyda Eruyar:** Supervision, Investigation. **Elijah Mironga Getanda:** Supervision, Investigation. **Sadiyya Haffejee:** Supervision, Investigation, Methodology. **Murali Krishna:** Supervision, Investigation. **Julian Edbrooke-Childs:** Conceptualization, Methodology, Funding acquisition.

## Declaration of Competing Interest

The authors declare that they have no known competing financial interests or personal relationships that could have appeared to influence the work reported in this paper.
